# Exploring Body Composition and Eating Habits Among Nurses in Poland

**DOI:** 10.3390/nu17162686

**Published:** 2025-08-20

**Authors:** Anna Bartosiewicz, Katarzyna Dereń, Edyta Łuszczki, Magdalena Zielińska, Justyna Nowak, Anna Lewandowska, Piotr Sulikowski

**Affiliations:** 1 Department of Health Sciences and Psychology, Collegium Medicum of Rzeszow University, 35-959 Rzeszów, Poland; kderen@ur.edu.pl (K.D.); eluszczki@ur.edu.pl (E.Ł.); mazielinska@ur.edu.pl (M.Z.); 2 Department of Cardiovascular Disease Prevention, Faculty of Public Health in Bytom, Medical University of Silesia, 41-902 Bytom, Poland; justyna.nowak@sum.edu.pl; 3 Faculty of Healthcare, State Academy of Applied Sciences in Jaroslaw, 37-500 Jarosław, Poland; am.lewandowska@poczta.fm; 4 Faculty of Computer Science and Information Technology, West Pomeranian University of Technology, 71-210 Szczecin, Poland; piotr.sulikowski@zut.edu.pl

**Keywords:** body composition, eating habits, BIA, nurses

## Abstract

**Background/Objectives**: Nurses play a vital role in healthcare, yet their demanding working conditions, including long hours, shift work, and stress, can negatively impact health behaviors. In Poland, empirical data on nurses’ eating habits and body composition remain limited. Therefore, this study aimed to evaluate body composition and dietary habits among nurses, and to identify significant relationships and associations between these variables. **Methods**: A cross-sectional observational study was conducted among 460 Polish nurses. The mean age of the respondents was 45.07 years (SD ± 11.98). Body composition was assessed using the Tanita MC-780 PLUS MA analyzer, and eating behaviors were measured with the standardized My Eating Habits questionnaire (MEH). Advanced statistical analyses including k-means clustering, ANOVA, chi-square tests, Spearman’s correlation, ROC curves, decision tree modeling, and heatmap visualization were used to identify associations. **Results**: The MEH scores among nurses indicated average eating behavior. However, excess body fat, overweight/obesity, shift work, and holding multiple jobs were significantly associated with emotional overeating, habitual overeating, and restrictive eating. Decision tree analysis identified Body Mass Index (BMI), fat-free mass (FFM) and comorbidities as key predictors of problematic eating patterns. Interaction effects showed that shift work combined with higher BMI further increased the risk of maladaptive behaviors. Heatmaps confirmed the strongest MEH scores in participants with elevated BMI and FFM. **Conclusions**: The findings underscore the need for targeted workplace interventions promoting healthy eating and weight control among nurses. Recognizing risk factors such as excess weight or multiple job holding can aid in designing effective prevention and health promotion strategies tailored to healthcare professionals.

## 1. Introduction

The global surge in overweight and obesity has escalated into a pressing public health emergency [1], prompting a coordinated response from the World Health Organisation (WHO) [2], the Organisation for Economic Co-operation and Development (OECD) [3], and national healthcare authorities [4]. According to the WHO Global Action Plan on the Prevention and Control of Noncommunicable Diseases (2013–2030), unhealthy diets and sedentary lifestyles are among the leading modifiable risk factors contributing to obesity, cardiovascular disease, type 2 diabetes, and other noncommunicable conditions that are placing an increasing burden on healthcare systems worldwide [2]. As the prevalence of these conditions continues to escalate, researchers and public health experts are increasingly focused on unraveling the primary factors influencing individuals’ body composition [1,5,6]. Among these factors, dietary habits emerge as a pivotal aspect, encompassing patterns and behaviors concerning meal selection, preparation, and consumption, which are deemed as fundamental determinants of calorie intake in terms of both quality and quantity [7]. Furthermore, environmental, social, and occupational factors exert a substantial influence on shaping these dietary habits [8,9].

Scientific investigations indicate that the demands of nursing work, including extended working hours, limited meal breaks, and high levels of stress, may contribute to unfavorable alterations in nurses’ dietary habits [10,11]. Suboptimal dietary practices, such as indulging in unhealthy snacks during shifts or resorting to convenience meals due to time constraints, can result in excessive calorie consumption and inadequate nutrient intake [12,13]. However, the potential influence of these factors on nurses’ body composition remains poorly understood, and the role of confounding variables such as age, gender, physical activity, and socio-economic status has not been sufficiently addressed. Consequently, these associations may lead to detrimental changes in body composition, elevating the risk of overweight, obesity, and related conditions like type 2 diabetes and cardiovascular diseases [11,12,13]. Given the current nursing shortage and the advancing age of nursing staff, with the average age surpassing 54 years, conducting research in this domain assumes particular significance [14,15]. According to the Supreme Chamber of Nurses and Midwives, the average age of practicing nurses in Poland has increased by over a decade in the past 20 years, with more than 35% of active nurses now above 55 years of age [14,15]. The OECD and WHO likewise emphasize that aging health workforces are a growing global concern, threatening the continuity and quality of care [16,17]. Simultaneously, empirical data indicate elevated rates of obesity, hypertension, and metabolic syndrome among aging nursing populations, conditions directly linked to irregular nutrition, stress, and shift work. Without timely intervention, these comorbidities contribute to early professional attrition, reduced work capacity, and rising healthcare costs [18].

Conducting focused research on occupational and dietary risk factors in this demographic is, therefore, not merely academic; it is a critical investment in public health resilience, workforce sustainability, and the safety of patients. Understanding how structural workplace stressors impact nurses’ physical health is essential to designing effective prevention strategies. These may include institutional meal reforms, health screenings, wellness programs, and policies that mitigate burnout and support healthy aging within this vital profession. Moreover, implementing strategies to safeguard the health of nurses represents a critical investment in public health, fostering the delivery of high-quality care and ensuring patient safety [18,19].

This study aims to evaluate how occupational factors such as irregular working hours, limited meal breaks, and high stress levels are associated with changes in body composition among nurses, while controlling for potential confounders. The central hypothesis is that these job-related stressors significantly impact nurses’ dietary habits and, consequently, their body composition. By comprehending the intricate interplay between dietary habits and body composition among nurses, more efficacious tailoring of health promotion strategies and preventive interventions can be developed to bolster the health and well-being of these vital healthcare professionals.

## 2. Materials and Methods

### 2.1. Participants and Study Design

This cross-sectional study, conducted between October 2023 and April 2024, involved 460 nurses employed in selected hospitals in the Subcarpathian region, following approval from the medical facility directors. Information regarding the measurements was disseminated to all nurses via the internal communication system. Interested participants could sign up on a prepared list, choosing a convenient date and time for the measurements.

To ensure the reliability of the data, strict inclusion and exclusion criteria were applied to participants, and any incomplete or incorrect answers were excluded from the analysis. The inclusion criterion was professionally active nurses willing to participate in measurements and surveys; exclusion criteria were nurses who were pregnant at the time and individuals with a pacemaker.

This study was approved by the Bioethics Committee of the University of Rzeszów (approval no. 2022/088) and conducted in accordance with the Declaration of Helsinki. All participants received verbal and written information and provided written informed consent before data collection.

The reporting of this cross-sectional study followed the STROBE (Strengthening the Reporting of Observational Studies in Epidemiology) guidelines. The completed STROBE checklist is available as an additional file named STROBE checklist.

The sample size was determined based on the estimated number of professionally active nurses in the Podkarpackie Voivodeship, which in 2023 included approximately 20,450 individuals (Central Register of Nurses and Midwives). The final sample comprised 460 participants, representing about 2.25% of the regional nursing population. The application of advanced statistical methods, including logistic regression, ROC analysis, cluster analysis, and decision tree modeling, allowed us to conclude that the sample size was sufficient to identify statistically and clinically relevant patterns.

### 2.2. Measurements

Participants underwent measurements of body weight and height using calibrated equipment and a standardized protocol. Body height was measured with participants standing upright without footwear, recorded to the nearest 0.1 cm using a portable stadiometer (Seca 213, Seca GmbH & Co. KG., Hamburg, Germany). Body mass was determined with a precision of 0.01 kg using a body composition analyzer (Tanita MC-980 PLUS MA, Tokyo, Japan) [20]. The measurements included body mass index (BMI), fat mass (FATM), fat-free mass (FFM), body fat percentage (FATP), and waist-to-hip ratio (WHR). Blood pressure was assessed using a validated oscillometric device, providing systolic blood pressure (SBP) and diastolic blood pressure (DBP). Normative ranges regarding body fat percentage were applied according to age as indicated by the body composition analyzer manufacturer:

Men (20–39 years):

<7.1% body fat below norm;
7.1–20.0% body fat within norm;
20.0–25.0% excessive body fat;
25.0% obesity.

Men (40 or more):

<10.1% body fat below norm;
10.1–22.0% body fat within norm;
22.1–28.4% excessive body fat;
28.4% obesity.

Women (20–39 years):

<20.9% body fat below norm;
20.9–33.0% body fat within norm;
33.1–39.5% excessive body fat;
39.5% obesity.

Women (40 or more):

<23.0% body fat below norm;
23.0–34.0% body fat within norm;
34.1–40.0% excessive body fat;
40.0% obesity [15].

BMI was calculated by dividing weight in kilograms by the square of height in meters (kg/m^2^). BMI categories were defined according to established recommendations: <16 = starvation; 16–16.99 = underweight; 17–18.49 = low weight; 18.5–24.99 = normal body weight; 25–29.99 = overweight; 30–34.99 = first-degree obesity; 35–39.99 = second-degree obesity; >40 = third-degree obesity [21].

Waist circumference was measured using an ergonomic anthropometric tape (Seca 201) between the lower edge of the rib cage and the upper iliac crest. Hip circumference was measured at the level of the iliac spine and the widest part of the buttocks. The waist-to-hip ratio (WHR) was calculated by dividing waist circumference by hip circumference.

For the WHR value, the following normative ranges were adopted:

Men:

<0.96 WHR within normal limits;
≥0.96 abdominal obesity, increased risk of metabolic diseases.

Women:

<0.83 WHR within normal limits;
≥0.83 abdominal obesity, increased risk of metabolic diseases [22].

### 2.3. Questionnaire

The survey aimed to evaluate the dietary habits of nurses using a standardized questionnaire, My Eating Habits (MEH), adapted for Polish conditions by Ogońska-Bulik and Putyński [23]. This questionnaire, designed for assessing dietary habits among adults, consists of 30 statements divided into three groups of factors diagnosing specific dietary habits. Participants responded to each statement with either “Yes” or “No,” with one point assigned for each “Yes” response. The cumulative points obtained allowed for determining the overall tendency of respondents towards inappropriate dietary habits, such as binge eating or food restriction. To differentiate between habitual and emotional binge eating, the results were separately calculated for three questionnaire factors, each containing 10 statements: Factor I—habitual overeating (0–10 points); Factor II—emotional overeating (0–10 points); and Factor III—tendency to restrict food intake (0–10 points). A diagnostic response was assigned “1” point, while a non-diagnostic response received “0” points. Diagnostic responses included “No” answers to statements 2, 5, 13, 17, 23, and “Yes” answers to the remaining statements.

The questionnaire facilitated the diagnosis of eating disorders and tendencies towards overweight, aiding in the selection of intervention strategies for reducing excessive body weight. The internal consistency of the questionnaire, measured by Cronbach’s alpha coefficient, was found to be 0.89 [23].

Additionally, the survey included inquiries regarding the socio-demographic profiles of participants, covering variables such as age, gender, educational attainment, job roles, work schedules, and engagement in supplementary employment activities. Administered in paper format alongside accompanying envelopes during the measurement sessions, nurses completed the surveys before returning them in sealed envelopes to the designated collection point. Ensuring anonymity, each measurement was coded, and every participant was assigned a distinct identification number to facilitate the correlation of survey responses with body composition outcomes.

### 2.4. Statistical Analysis Applied

Descriptive statistics, including mean, standard deviation, minimum, maximum, and quartiles, were calculated for all variables. The Kolmogorov–Smirnov test with Lilliefors correction was used to assess the normality of the distributions. Spearman’s rank correlation test was applied to evaluate the strength and direction of relationships between variables. To compare the groups, the Mann–Whitney U test was used to identify significant differences between independent groups. Logistic regression analysis was conducted to investigate the relationships between independent variables and the dependent variable, with Wald tests used to evaluate the statistical significance of the regression coefficients.

Cluster Analysis: Using the k-means method identified three distinct clusters for each of the factors assessed in the MEH Questionnaire, as well as the overall score. ANOVA and pairwise comparisons confirmed significant differences (*p* < 0.0001) between all pairs of analyzed variables.

Chi-Square Test: To identify the variables influencing the clustered results related to dietary habits, the chi-square test of independence (χ^2^) was applied. This method determined the relationships between categorical variables.

Correlation Analysis: Spearman’s rank correlation test was used to examine correlations between quantitative variables and the results of the MEH Questionnaire. Since the correlations were weak, the results were reported as odds ratios (OR) to better illustrate the relationships. The odds ratios were calculated to assess the strength and direction of associations between specific independent variables and the outcome variables.

ROC (Receiver Operating Characteristic) analysis: For variables that were not initially categorized (such as age, FFM, and blood pressure), binary variables were created through ROC analysis, taking into account the “gender” variable. The cutoff point for grouping was established (<cutoff point: group 1, ≥cutoff point: group 2).

Decision Tree Analysis: For each factor, a separate decision tree was created to identify which variables significantly differentiate that factor. This helped visualize the most influential variables for each outcome.

Heatmap Visualization: The intensity of each factor, according to the variables extracted from the decision trees, was visualized using heatmaps. The color intensity indicates the concentration of a particular characteristic within each group, with darker colors representing higher levels of the feature.

The regression model was adjusted for a predefined set of demographic and professional covariates, including age, gender, education level, years of experience, work setting, and employment type. To enhance interpretability, only results for the “high” category are presented in the main tables of the manuscript. Full model specifications and detailed results for all outcome levels are provided in Part II of the Supplementary Materials.

A significance level of *p* < 0.05 was adopted for all analyses. All calculations were performed using Statistica software, version 13.0.

## 3. Results

### 3.1. Characteristics of Study Group

The study group consisted of 460 nurses (420 women and 40 men). The average age of the respondents was 45.07 years (SD ± 11.98). The vast majority (83.9%) worked in hospital wards. More than half (51.5%) worked in shifts, worked full-time (70.7%) and had completed higher education (*n* = 104; 22.6%, *n* = 218; 47.4%) (i.e., completed a bachelor’s or master’s degree in nursing). Detailed characteristics are presented in Table 1.

### 3.2. Findings

Table 2 presents descriptive statistics illustrating the distribution and variability of selected anthropometric and hemodynamic parameters among study participants. The analyzed physical characteristics include BMI, FATP, FATM, FFM, WHR, SBP, and DBP.

For each parameter, the following descriptive statistics are reported: the mean (M) along with the 95% confidence interval (CI), the median (Me) with interquartile range (IQR; Q1–Q3), and the standard deviation (SD) including minimum and maximum values. This comprehensive presentation provides an overview of the central tendency, dispersion, and distribution range of each variable, offering a robust understanding of the sample’s physical and cardiovascular profile.

The results revealed noticeable variability in the average scores and distribution across the three dimensions of the My Eating Habits questionnaire, indicating differing intensities of eating behaviors within the study group. Habitual overeating (Factor 1) and emotional overeating (Factor 2) demonstrated similar mean values and standard deviations, suggesting a comparable level of occurrence in the surveyed population. In contrast, dietary restrictions (Factor 3) showed slightly lower scores, reflecting less frequent engagement in restrictive eating behaviors. The overall score, calculated as the sum of all three MEH factors, ranged broadly from 0 to 28 points, suggesting diverse dietary patterns among the participants (Table 3).

K-means cluster analysis was performed to categorize participants based on their responses to the three factors of the My Eating Habits questionnaire, as well as the overall dietary habit score. For each variable, three distinct clusters were identified: low, medium, and high levels of behavior intensity.

Analysis of variance (ANOVA), followed by post hoc pairwise comparisons, revealed statistically significant differences between the clusters (*p* < 0.0001) across all factors, confirming the existence of distinct patterns of eating behavior within the study population. The most common cluster for emotional overeating was the low-level group (51.5%), while medium and low levels were predominant for habitual overeating and dietary restrictions, respectively. These findings suggest that, while some participants show elevated tendencies in specific eating behaviors, the majority present with moderate or lower intensity patterns, particularly in emotional and restrictive eating (Table 4).

Chi-square (χ^2^) independence tests were conducted to examine the relationships between sociodemographic and health-related variables and the clustered levels of overall dietary habits. The analysis revealed that certain variables significantly influenced the distribution of dietary habit clusters. Specifically, higher overall dietary habit scores were significantly more common among participants working more than one job (*p* = 0.0170), with overweight or obesity (*p* = 0.0010), and reporting the presence of chronic diseases (*p* = 0.0295). No statistically significant associations were observed for sex, type of work, work system, or WHR (Table 5). Detailed statistical results supporting these findings are provided in Part I of the Supplementary Material (Tables S1–S3).

### 3.3. Decision Tree Analysis of Eating Behaviors

To better identify which variables significantly influence specific eating behavior patterns, separate decision tree analyses were conducted for each factor of the My Eating Habits questionnaire, namely habitual overeating (Factor 1), emotional overeating (Factor 2), dietary restrictions (Factor 3), and the overall dietary habit score. The decision trees were constructed using the CART (Classification and Regression Tree) method. Input variables included age, sex, FFM, SBP, DBP, BMI, FATP, employment pattern (including shift work and multiple jobs), and presence of chronic diseases. For continuous variables, binary cut-off points were established using ROC curve analysis to enhance model interpretability.

**Figure 1:**
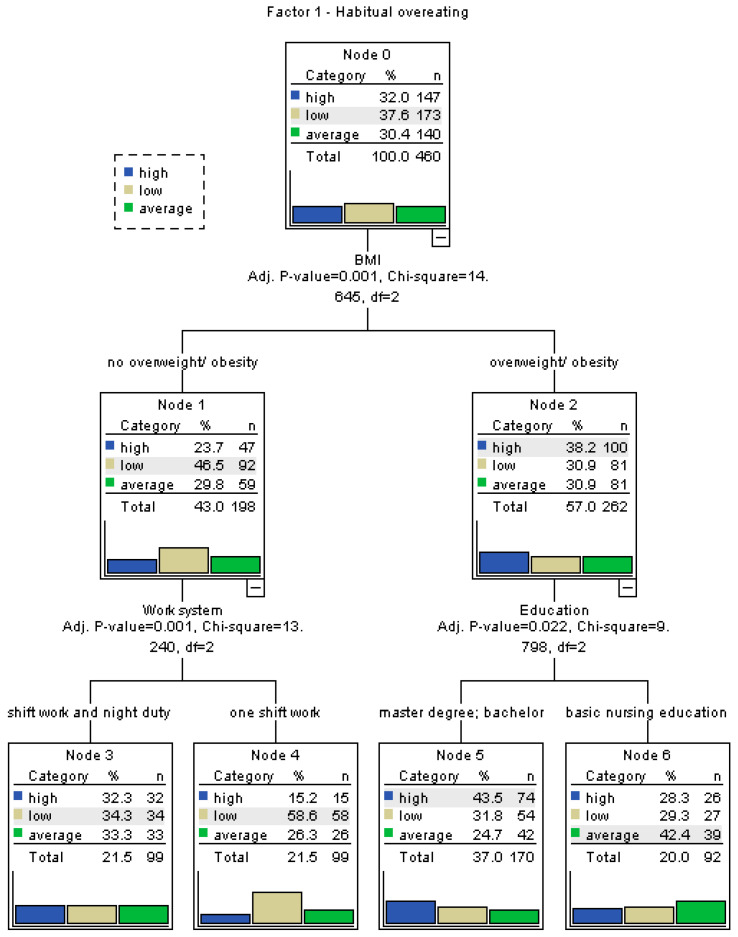
Decision tree for Factor 1—habitual overeating. Habitual overeating was most commonly associated with nurses working shifts, holding multiple jobs, and presenting with elevated FATP and BMI.

**Figure 2:**
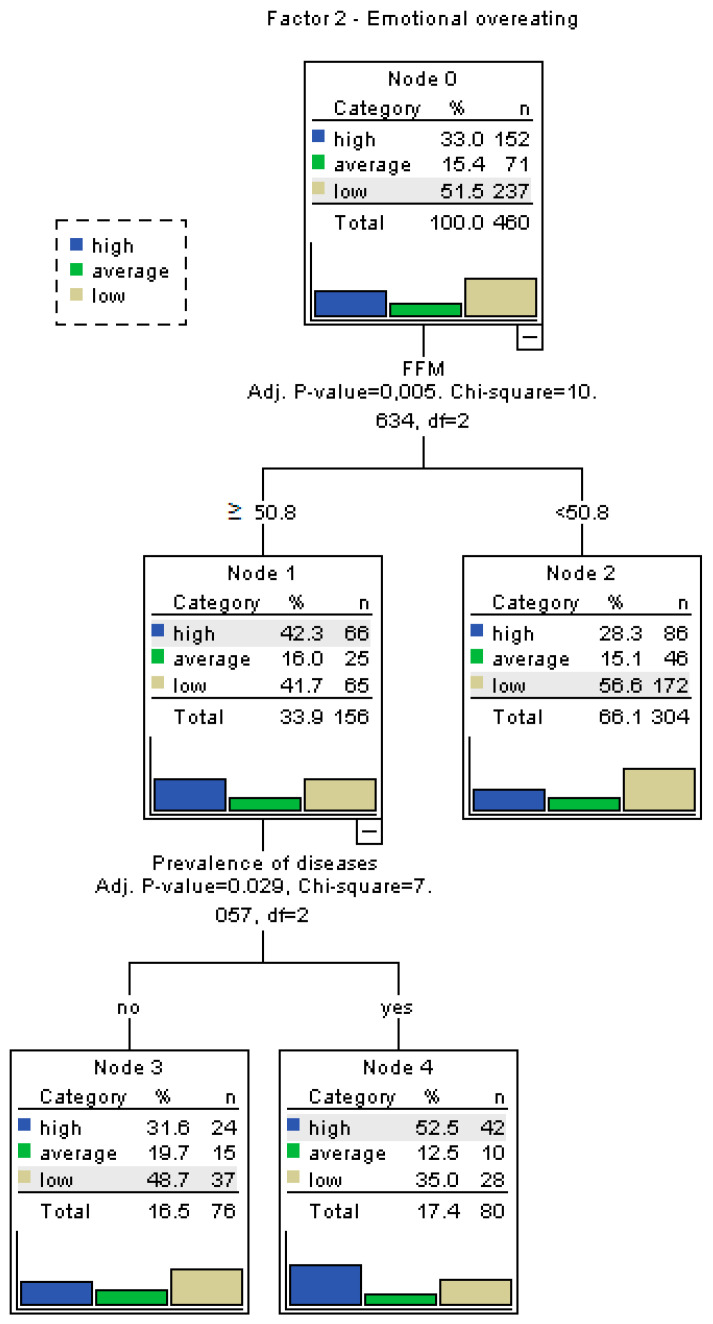
Decision tree for Factor 2—emotional overeating. The primary determinant was BMI, with higher emotional overeating observed in overweight or obese participants.

**Figure 3:**
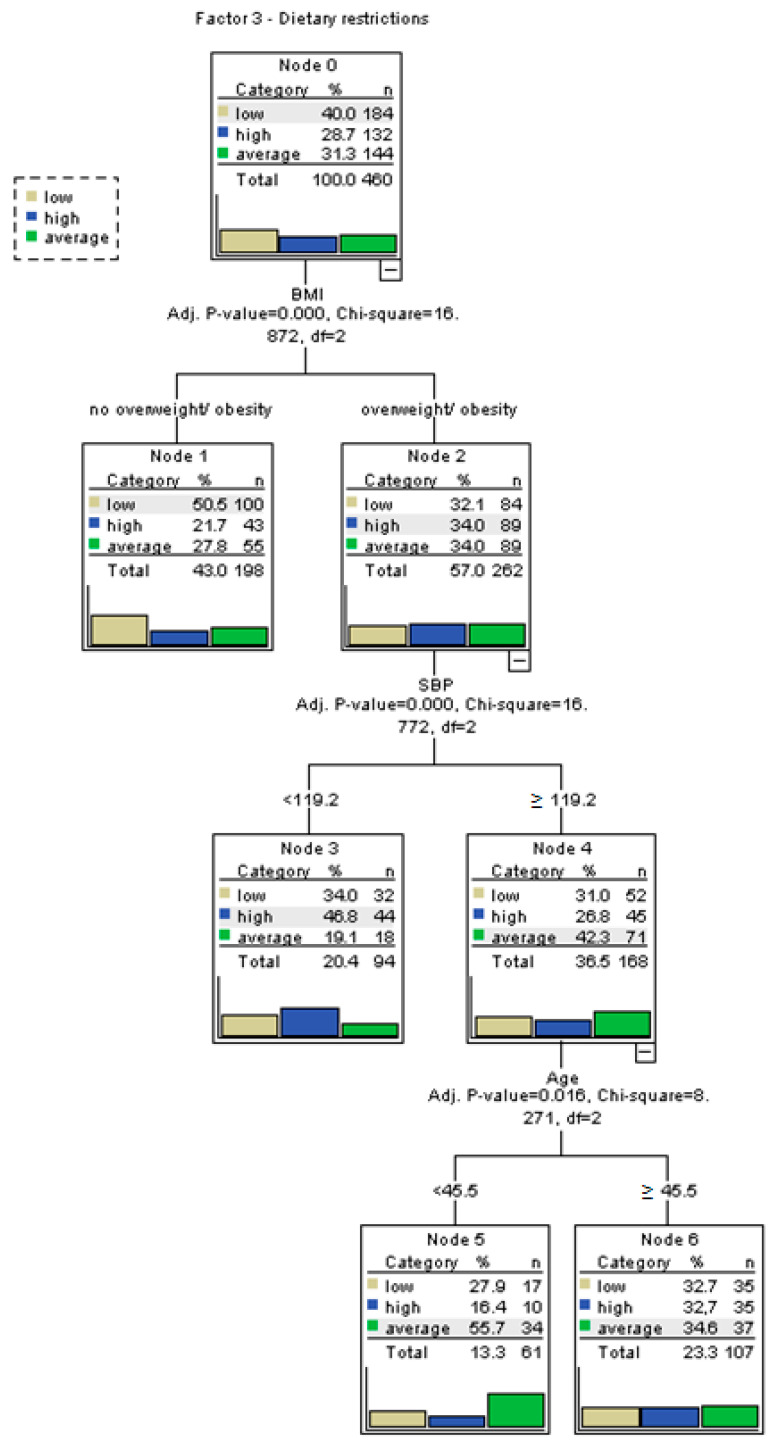
Decision tree for Factor 3—dietary restrictions. Dietary restriction was more likely in individuals with increased body fat percentage and BMI.

**Figure 4:**
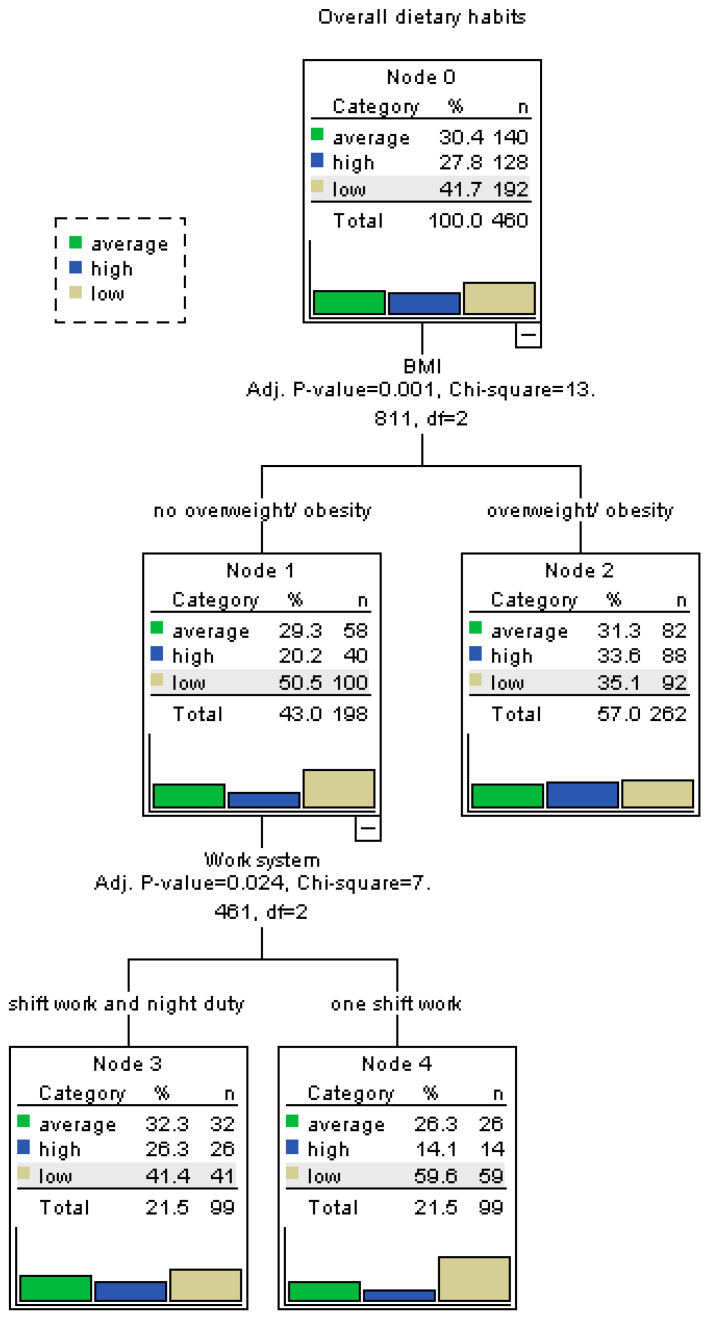
Decision tree for overall dietary habits. Variables such as age, fat-free mass, and blood pressure significantly contributed to the differentiation of dietary behavior.

For the factor “Habitual overeating”, high scores were more frequently observed among individuals working in a two-shift system (*p* = 0.0011), working more than one job (*p* = 0.0085), having increased body fat (*p* = 0.0495), being overweight or obese (*p* = 0.0007), and having comorbidities (*p* = 0.0316). As shown in Figure 1, habitual overeating was most prevalent among participants meeting these combined criteria. In the case of “Emotional overeating”, high scores were significantly associated with participants’ BMI (*p* = 0.0113), with elevated results more common among those classified as overweight or obese. Figure 2 illustrates BMI as the main differentiator in the model, with minimal input from other variables. For “Dietary restrictions”, the most influential predictors were body fat percentage and BMI. High scores were more frequent among individuals with increased body fat (*p* = 0.0249) and those classified as overweight/obese (*p* = 0.0002), as seen in Figure 3. The decision tree for the overall dietary habit score incorporated a broader range of variables. Age, FFM, SBP, and DBP, all divided into binary categories based on ROC-determined thresholds, significantly contributed to the classification. Figure 4 demonstrates how both physiological (blood pressure, body composition) and demographic (age) variables influence general eating behavior patterns (Figure 1, Figure 2, Figure 3 and Figure 4).

The ROC-based binary divisions were as follows: 44.8% of participants were younger than 45.5 years, 66.1% had a fat-free mass (FFM) below 50.8 kg, 43.5% had a systolic blood pressure (SBP) below 119.2 mmHg, and 39.6% had a diastolic blood pressure (DBP) below 73.8 mmHg. Additionally, the “sex” variable was categorized into two groups based on an ROC-derived cut-off point, enabling further analysis of potential gender-related patterns. Detailed results of the ROC analysis are presented in Table 6.

The visualization of the intensity of a given factor according to variables identified through decision trees is presented using heatmaps. The more intense the color, the higher the intensity of the trait in a given group. For the first decision tree, the included variables are BMI and work system or BMI and education, as these variables proved to be the most important in assessing Factor 1 among all others.

### 3.4. Heatmaps of Eating Behavior Factors by Key Variables

**Figure 5:**
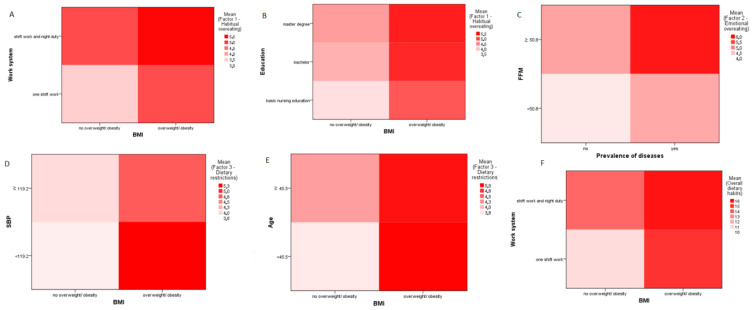
Heatmaps illustrating the intensity of eating behavior dimensions across sociodemographic and clinical subgroups: (**A**) Habitual overeating by BMI and work system, (**B**) Habitual overeating by BMI and educational level, (**C**) Emotional overeating by FFM and prevalence of diseases, (**D**) Dietary restriction by BMI and SBP, (**E**) Dietary restriction by BMI and age, (**F**) Overall dietary habits by BMI and work system.

This figure presents six heatmaps illustrating the mean scores for selected eating behavior dimensions based on the My Eating Habits questionnaire. The color intensity reflects the magnitude of the respective behavior, with darker shades indicating higher values. Each map combines two categorical variables identified through decision tree analysis to explore how sociodemographic and clinical factors interact in shaping dietary behaviors among nurses (Figure 5).

Odds ratios (ORs) were estimated using logistic regression analysis. The results indicated that high scores for Factor 1 (habitual overeating) were significantly more prevalent among nurses working in a two-shift system (OR = 1.58; *p* = 0.0247), those employed in more than one job (OR = 1.91; *p* = 0.0025), individuals with increased body fat (OR = 1.74; *p* = 0.0244), those classified as overweight or obese (OR = 1.98; *p* = 0.0011), participants with comorbid conditions (OR = 1.67; *p* = 0.0111), and those with an FFM ≥ 50.8 kg (OR = 1.70; *p* = 0.0106). High scores for Factor 2 (emotional overeating) were more common among participants who were overweight or obese (OR = 1.81; *p* = 0.0041) and those with elevated FFM (OR = 1.86; *p* = 0.0026). In the case of Factor 3 (dietary restrictions), higher scores were more frequently associated with excessive body fat (OR = 1.71; *p* = 0.0314), overweight/obesity (OR = 1.85; *p* = 0.0043), and age ≥ 45.5 years (OR = 1.56; *p* = 0.0367).

For the overall MEH score, high values were observed more often among nurses working shifts (OR = 1.55; *p* = 0.0370), those holding multiple jobs (OR = 1.86; *p* = 0.0048), individuals with excess body fat (OR = 1.71; *p* = 0.0332), those classified as overweight or obese (OR = 2.00; *p* = 0.0017), and those with chronic diseases (OR = 1.68; *p* = 0.0133) (Table 7). Additional odds ratios for low and medium values of eating behaviors are presented in Part II of the Supplementary Material (Tables S1–S4).

## 4. Discussion

This study revealed that Polish nurses demonstrate moderate levels of various maladaptive eating behaviors, particularly habitual and emotional overeating, with significant associations identified between these behaviors and BMI, work schedule (especially double shifts), and comorbidities. Younger age was not a protective factor, and high-risk subgroups were identified via CART analysis, notably including nurses with higher FFM and overweight/obesity and those holding multiple jobs. Emotional eating and dietary restriction were especially prevalent among those with higher BMI and elevated blood pressure, suggesting a physiological–emotional interplay in maladaptive eating behaviors. These findings suggest that job-related stressors and metabolic burden collectively shape dietary patterns in this population.

Nurses’ dietary habits have garnered increasing attention in public health literature due to their significant implications for both individual well-being and professional performance [24]. Nurses’ nutritional status directly affects energy levels, immune function, psychological resilience, and the capacity to provide optimal patient care [25].

The present study employed a multi-method design using the MEH questionnaire, decision trees, logistic regression, and heatmaps, allowing a granular analysis of dietary behavior predictors.

Many studies underscore the significance of attending to the development of proper dietary practices, as they play a pivotal role in maintaining health and well-being [26,27,28]. This is particularly crucial for healthcare professionals, notably nurses, whose daily occupational demands can induce stress and overwhelm [29]. A growing body of research confirms that poor nutrition among nurses negatively affects not only their metabolic health but also their psychological well-being and clinical performance [30]. Adopting correct dietary practices and adhering to a balanced diet are the factors that influence the body’s immunity, a matter deserving special attention given the taxing nature of nurses’ work conditions [31,32,33]. Adhering to a balanced diet can not only enhance overall health but also bolster energy levels, focus, and workplace efficacy [34,35].

Our data align with prior findings, confirming that nurses with overweight or obesity are more prone to habitual and emotional overeating [24,36,37]. Geniusz-Wojczyk et al. observed emotional overeating as the predominant eating habit among the surveyed nurses. Furthermore, overweight/obese nurses, part-time workers, and younger nurses displayed a propensity for making poor dietary choices, including overeating or food restriction [38].

While previous literature has consistently emphasized the high prevalence of poor dietary behaviors among nurses due to shift work and occupational stress [24,25], our study expands this understanding by identifying specific work-related and metabolic risk factors, and showing how these persist after adjusting for demographic variables. These associations persisted even after adjusting for sex, age, and education level, confirming the robustness of occupational and metabolic predictors in this population.

The prevalence of unfavorable eating habits among nurses, particularly emotional overeating, constitutes a multifaceted issue influenced by various factors. The analysis highlighted how factors like shift work, multiple job holding, and higher FFM contributed to unhealthy eating habits. These variables appear repeatedly across our trees, suggesting robust and consistent associations. This resonates with literature emphasizing the disruptive role of circadian misalignment caused by shift work on appetite regulation, metabolic hormones, and emotional coping strategies [38,39,40].

Several researchers attribute this trend to the stressful and demanding nature of nursing work, exacerbated by shift schedules that disrupt natural metabolic rhythms, potentially leading to maladaptive eating behaviors such as emotional overeating [41,42]. However, it is important to critically assess whether these maladaptive eating behaviors are solely due to work-related stress or if there are other contributing factors, such as personal predispositions or socio-economic conditions, that might play a more substantial role. Brzeźniak highlights workplace stress as a contributing factor to nutrition-related disorders among nurses, with 30% reporting occasional eating disorders and abdominal discomfort following shifts [43]. Nurses’ dietary patterns are intertwined with workload and time constraints, often resulting in the consumption of quick, simple, and cold meals. Warchoł-Satwińska notes that nurses’ eating habits are largely dictated by immediate needs and financial considerations [44]. Time constraints also prompt reliance on snacks, with 70% of snacks being unhealthy choices like chocolate, chips, and sugary beverages, while only 30% consist of healthier options such as fruits, vegetables, yogurt, and nuts [45]. Monaghan et al. identified another significant challenge affecting nurses’ dietary habits: the prevalence of high-sugar, high-fat foods donated by patients’ families, readily available in break rooms [46].

While these factors are important, the role of institutional policies, such as the availability of nutritious meals or the regulation of snack options, should also be considered as potential moderators of dietary behaviors.

The impact of job structure is further supported by odds ratio analysis, which revealed that nurses working more than one job were nearly twice as likely to engage in habitual overeating (OR = 1.91; *p* = 0.0025), and those with comorbidities also showed elevated risk of maladaptive dietary behaviors. These findings may suggest a cycle in which occupational demands reinforce poor dietary choices, contributing to further health deterioration. A study conducted among nurses in Saudi Arabia demonstrated that those who seldom ate breakfast and frequently consumed fast food were more likely to be overweight or obese [47]. Ogińska-Bulik and Putyński’s research indicated that overweight women exhibited a higher propensity for emotional and habitual overeating, as well as more frequent restrictive dietary behaviors, compared to women of normal weight [23]. Irregular meal patterns, stemming from the demands of nursing duties, contribute to poor eating habits. Nurses often forego meals for extended periods, leading to intense feelings of hunger and dehydration [48,49]. Moreover, the fast-paced and unpredictable nature of their work inhibits the opportunity to sit down and eat leisurely, impacting the type and quantity of food consumed [50]. BMI demonstrates a significant and positive correlation with restrained eating, emotional eating, and stress. Eating hastily and encountering numerous stressful situations may trigger uncontrollable eating and subsequent weight gain [51,52]. Moreover, nurses who work shifts and night shifts demonstrate a higher tendency toward overeating compared to those on single shifts, likely due to differences in lifestyle rhythms and challenges in maintaining regular meals during non-traditional working hours [53]. Samhat et al. found that nearly 80% of nurses reported irregular meal patterns during the day and increased snacking during night shifts. Shift work correlated positively with abnormal eating behaviors and BMI among Lebanese nurses [54]. According to Bilski, shift work also influences the quality of meals consumed by nurses, often limited to cold meals and coffee. Their diets frequently consist of difficult-to-digest, calorically dense foods rich in preservatives, with only a minority consuming hot meals during night shifts [55,56]. Similarly, Nejman and Gotlib observed a high prevalence of dietary mistakes among nurses, with diets characterized as merely sufficient. A majority admitted to paying little attention to the types of products and meals consumed [57].

Decision tree analysis highlighted BMI, FFM, and comorbidities as the most consistent predictors of high MEH scores, particularly in the domains of emotional overeating and dietary restriction. The trees also illustrated non-linear interactions, suggesting that the combination of shift work and overweight/obesity amplifies the risk of unhealthy eating. Additionally, heatmaps showed that emotional overeating and dietary restriction behaviors were more pronounced among those with higher BMI, elevated SBP and DBP, and increased FFM. This may point to compensatory eating as a maladaptive response to physiological strain, echoing psychosomatic theories of eating behavior [58].

Notably, while younger age was previously thought to be a risk factor, our results show high scores across age categories, suggesting that metabolic and job-related stressors may override age as a determinant. Interestingly, while BMI was consistently significant across all models, systolic and diastolic blood pressure did not emerge as independent predictors, indicating that cardiovascular dysregulation may lag behind behavioral symptoms in this context. Our findings underline the complexity of the issue, where not only the work schedule and time constraints but also dietary habits linked to emotional responses and stress contribute to the deterioration of nurses’ health. It is crucial to recognize the interdependence of these factors and how they collectively influence nurses’ well-being. Moreover, our results support the growing emphasis on institutional responsibility, as current workplace environments, including a lack of structured breaks, limited access to healthy meals, and sociocultural acceptance of unhealthy snacking, are likely contributing to this public health burden [53,59,60].

The purpose of this study was to investigate how work conditions, metabolic strain, and emotional factors interact to shape dietary behaviors in Polish nurses. The results offer valuable insights for occupational health, showing that specific risk profiles, such as multiple job holding and high BMI, can guide the development of targeted interventions. By triangulating advanced statistical methods and validated tools, this study contributes to a more nuanced understanding of psychosomatic eating patterns among healthcare professionals. Future research should build upon these findings using longitudinal and interventional designs.

This study has several limitations that must be acknowledged. The sample was drawn from a single region in Poland, limiting generalizability to other geographical, cultural, and institutional contexts. Dietary behaviors and occupational conditions may differ substantially across healthcare systems; thus, findings may not be representative of the wider nursing population. Also, the MEH scale, while validated, relies on self-report and may be subject to bias, including social desirability and recall bias, particularly when reporting eating behaviors related to emotional states. Furthermore, the cross-sectional design precludes causal inference, making it impossible to determine whether occupational or metabolic factors preceded the development of maladaptive eating behaviors, or whether these behaviors contributed to the observed physiological parameters.

In addition, the study did not account for several potentially influential variables, such as socioeconomic status, dietary knowledge, access to food at the workplace, or psychological distress, all of which may confound the observed associations. Moreover, the assessment did not include objective nutritional data (e.g., food diaries, calorie intake, or nutrient composition), which would have provided a more precise characterization of dietary patterns. Some variables, such as blood pressure or FFM, while measured objectively, may still fluctuate depending on short-term conditions (e.g., shift fatigue), possibly influencing interpretation.

Lastly, the statistical models used, although robust, may not capture the full complexity of dynamic and non-linear interactions over time. Longitudinal or experimental designs would be better suited to establish directionality and explore causality in the observed associations. Despite these limitations, the use of advanced modeling and a large sample size enhance the reliability of the current findings. Nevertheless, interpretations should remain cautious, and future studies should aim to address these shortcomings.

Despite the above, the study provides valuable insights into the multifactorial nature of dietary behaviors among nurses, and its methodological rigor ensures that the findings remain robust within the defined context. However, the generalizability of these findings may be limited to similar populations, particularly professionally active nurses working in comparable healthcare systems and organizational environments

In summary, our study contributes novel empirical insight by triangulating multiple quantitative analyses to map the psychosocial and physiological dimensions of eating behavior in Polish nurses. The study findings reveal that eating habits among nurses, as assessed by the MEH questionnaire, are moderate for each factor. Statistically significant correlations were observed between BMI and various eating behaviors, including habitual overeating, emotional overeating, dietary restrictions, and overall eating habits. Moreover, significant relationships were identified between overall eating habits and factors such as habitual overeating and double-shift work, as well as emotional overeating and dietary restrictions concerning overweight and obesity.

In light of these findings, the importance of prioritizing nurses’ healthcare, particularly within the context of the conditions prevailing in our country, cannot be overstated. Recent data from the Supreme Chamber of Nurses and Midwives reveals a distressing reality: the average lifespan of nurses is approximately 20 years shorter than that of the general female population in our country. It is paradoxical that those who dedicate themselves professionally to patient care experience such a significant disparity in life expectancy compared to their peers [61]. This discrepancy is largely attributed to overwork, shift work, and the lack of time to prioritize a healthy lifestyle and personal health [62].

## 5. Conclusions

These findings carry significant implications for clinical practice and health management. Implementing workplace programs aimed at promoting healthy eating and encouraging employees to maintain a healthy weight can be pivotal in improving nurses’ dietary behaviors and overall health. Furthermore, identifying risk factors such as working multiple jobs or abnormal body weight can inform targeted preventive and intervention strategies aimed at enhancing the health outcomes of medical workers. Overall, this study has the potential to inform the development of preventive and intervention initiatives focused on improving nurses’ eating habits and overall health. By narrowing the life expectancy gap between nurses and the general female population and enhancing health outcomes among healthcare workers, these efforts can contribute to a healthier workforce and improved patient care. Future longitudinal and intervention-based studies are warranted to assess whether organizational changes (e.g., meal programs, psychological support, time management workshops) can mitigate these negative trends.

Future research should prioritize longitudinal and interventional designs to explore how occupational, metabolic, and psychological factors jointly influence eating behaviors among nurses. Interdisciplinary collaboration involving public health, occupational medicine, and behavioral nutrition is essential for designing sustainable dietary health policies for healthcare workers.

## Figures and Tables

**Table 1:** Characteristics of the study group.

Parameter		Total (*n* = 460)	
		*n*	%
Sex *	female	420	91.3%
	male	40	8.7%
Type of work *	hospital ward	386	83.9%
	administrative position	74	16.1%
Work system *	one shiftwork	223	48.5%
	shift work and night duty	237	51.5%
More than one job *	No	325	70.7%
	Yes	135	29.3%
Education *	basic nursing education	138	30.0%
	bachelor	104	22.6%
	master’s degree	218	47.4%
WHR *	normal	243	52.8%
	abdominal obesity	217	47.2%
BMI *	underweight	8	1.7%
	normal body weight	190	41.3%
	overweight	154	33.5%
	first-degree obesity	72	15.7%
	second-degree obesity	23	5.0%
	third-degree obesity	13	2.8%
Fat mass *	normal	325	70.7%
	excessive body fat	91	19.8%
	obesity	44	9.6%
Prevalence of diseases *	No	237	51.5
	Yes	223	48.5
Prevalence of diseases *	diabetes	29	6.3
	hypertension	69	15.0
	circulatory system diseases	20	4.3
	thyroid diseases	63	13.7
	kidney disease	10	2.2
	digestive system diseases	22	4.8
	degenerative disease of joints/spine	48	10.4
	diseases of the nervous system	7	1.5
	cancer	6	1.3
	eating disorders: anorexia	4	0.9
	bulimia	8	1.7
	compulsive overeating syndrome	22	4.8
	other	45	9.8

* Data presented as: *n* (%).

**Table 2:** Anthropometric measurements in the study group.

Parameter	M (95% CI)	Me (Q1–Q3)	SD (Min–Max)
BMI [kg/m^2^]	26.77 (26.28; 27.26)	25.8 (23.1; 29.5)	5.39 (16.6; 54.6)
FATP [%]	30.29 (29.62; 30.96)	30.4 (25.35; 35.3)	7.34 (10; 52.4)
FATM [kg]	22.58 (21.67; 23.49)	20.85 (16; 26.85)	9.9 (4.6; 75.7)
FFM [kg]	49.43 (48.7; 50.15)	47.9 (44; 53.1)	7.88 (32.4; 80.8)
WHR [cm]	0.83 (0.83; 0.84)	0.83 (0.78; 0.89)	0.08 (0.52; 1.10)
SBP [mmHg]	124.08 (121.99; 126.17)	122 (111.75; 132.00)	22.81 (90.67; 198.00)
DBP [mmHg]	77.45 (75.62; 79.29)	75.83 (71; 81)	20.04 (56.67; 118.00)

M—mean (average) value for each physical characteristic; CI—confidence interval; SD—standard deviation, a measure of the dispersion or spread of the data; Min—minimum value observed for each characteristic; Max—maximum value observed for each characteristic; Me—median; Q1–Q3—the first quartile and third quartile (Q3) of the data, BMI—Body Mass Index; WHR—Waist/Hip Ratio; FATP—Fat Adipose Tissue Percentage; FATM—Fat Adipose Tissue Mass; FFM—Fat-Free Mass; SBP—systolic blood pressure; DBP—diastolic blood pressure.

**Table 3:** Nurses’ eating habits divided into factors of the My Eating Habits questionnaire.

Factors of MEH Questionnaire	M (95% CI)	Me (Q1–Q3)	SD (Min–Max)
Factor 1—Habitual overeating	4.66 (4.42; 4.9)	4 (3; 7)	2.6 (0; 10)
Factor 2—Emotional overeating	4.71 (4.46; 4.96)	4.5 (3; 7)	2.76 (0; 10)
Factor 3—Dietary restrictions	4.47 (4.28; 4.67)	4 (3; 6)	2.17 (0; 10)
Overall dietary habits	13.85 (13.28; 14.41)	13 (9; 18)	6.15 (0; 28)

M—mean (average) value for each physical characteristic; CI—confidence interval; SD—standard deviation, a measure of the dispersion or spread of the data; Min—minimum value observed for each characteristic; Max—maximum value observed for each characteristic; Me—median; Q1–Q3—the first quartile (Q1) and third quartile (Q3) of the data.

**Table 4:** Cluster analysis of eating habits based on the MEH questionnaire.

Factors of MEH Questionnaire	Level of Clusters	*n*	%
Factor 1—Habitual overeating	High	147	32.0
	Low	173	37.6
	Medium	140	30.4
Factor 2—Emotional overeating	High	152	33.0
	Medium	71	15.4
	Low	237	51.5
Factor 3—Dietary restrictions	Low	184	40.0
	High	132	28.7
	Medium	144	31.3
Overall dietary habits	Medium	140	30.4
	High	128	27.8
	Low	192	41.7

**Table 5:** Variables influencing eating habits in the studied group of nurses *.

Variables		Overall Dietary Habits [MEH]						*p*
		Average		High		Low		
		*n*	%	*n*	%	*n*	%	
Sex	female	129	30.7%	118	28.1%	173	41.2%	Χ^2^ = 0.598; *p*(χ^2^) = 0.7415
	male	11	27.5%	10	25.0%	19	47.5%	
Type of work	hospital ward	113	29.3%	107	27.7%	166	43.0%	χ^2^ = 1.992; *p*(χ^2^) = 0.3693
	administrative position	27	36.5%	21	28.4%	26	35.1%	
Work system	shiftwork	73	32.7%	52	23.3%	98	43.9%	χ^2^ = 4.418; *p*(χ^2^) = 0.1098
	shift work and night duty	67	28.3%	76	32.1%	94	39.7%	
More than one job	no	103	31.7%	78	24.0%	144	44.3%	χ^2^ = 8.152; *p*(χ^2^) = 0.0170
	yes	37	27.4%	50	37.0%	48	35.6%	
Education	basic nursing education	40	29.0%	30	21.7%	68	49.3%	χ^2^ = 7.540; *p*(χ^2^) = 0.1100
	Bachelor	31	29.8%	37	35.6%	36	34.6%	
	master’s degree	69	31.7%	61	28.0%	88	40.4%	
WHR	normal	75	30.9%	63	25.9%	105	43.2%	χ^2^ = 0.967; *p*(χ^2^) = 0.6168
	abdominal obesity	65	30.0%	65	30.0%	87	40.1%	
BMI	normal	99	30.5%	81	24.9%	145	44.6%	χ^2^ = 6.003; *p*(χ^2^) = 0.1989
	increased	28	30.8%	33	36.3%	30	33.0%	
	excessive	13	29.5%	14	31.8%	17	38.6%	
BMI	no overweight/obesity	58	29.3%	40	20.2%	100	50.5%	χ^2^ = 13.811; *p*(χ^2^) = 0.0010
	prevalence overweight/obesity	82	31.3%	88	33.6%	92	35.1%	
Prevalence of disease	no	73	30.8%	54	22.8%	110	46.4%	χ^2^ = 7.046; *p*(χ^2^) = 0.0295
	yes	67	30.0%	74	33.2%	82	36.8%	

* The table presents results for overall dietary behavior (MEH), while the findings for Factor I (habitual overeating), Factor II (emotional overeating), and Factor III (dietary restriction) are presented in Supplementary Material—Part I.

**Table 6:** Participant distribution according to cut-off points identified by ROC analysis for age, FFM, SBP, and DBP.

ROC		*n*	%
Age (AUC = 0.546 sensitivity = 0.567 1-specificity = 0.400)	<45.5	206	44.8%
	≥45.5	254	55.2%
FFM (AUC = 0.839 sensitivity = 0.875 1-specificity = 0.288)	<50.8	304	66.1%
	≥50.8	156	33.9%
SBP (AUC = 0.698 sensitivity = 0.875 1-specificity = 0.533)	<119.2	200	43.5%
	≥119.2	260	56.5%
DBP (AUC = 0.662 sensitivity = 0.875 1-specificity = 0.579)	<73.8	182	39.6%
	≥73.8	278	60.4%

SBP—systolic blood pressure; DBP—diastolic blood pressure; FFM—Fat-Free Mass; ROC—Receiver Operating Characteristic; AUC—Area Under the Curve.

**Table 7:** Odds ratios for high values of eating habits in the studied group *.

Variables	F1_High	F2_High	F3_High	MEH Overall
Sex	OR = 0.79 (0.38; 1.63); *p* = 0.5278	OR = 0.56 (0.26; 1.21); *p* = 0.1424	OR = 0.60 (0.27; 1.33); *p* = 0.2075	OR = 0.85 (0.40; 1.80); *p* = 0.6766
Type of work	OR = 0.95 (0.56; 1.63); *p* = 0.8601	OR = 0.90 (0.53; 1.54); *p* = 0.6953	OR = 0.98 (0.57; 1.70); *p* = 0.9475	OR = 1.03 (0.59; 1.79); *p* = 0.9079
Work system	OR = 1.58 (1.06; 2.34); *p* = 0.0247	OR = 1.41 (0.95; 2.09); *p* = 0.0854	OR = 1.19 (0.79; 1.78); *p* = 0.4106	OR = 1.55 (1.03; 2.35); *p* = 0.0370
More than one job	OR = 1.91 (1.25; 2.90); *p* = 0.0025	OR = 1.07 (0.70; 1.63); *p* = 0.7620	OR = 1.51 (0.98; 2.32); *p* = 0.0623	OR = 1.86 (1.21; 2.87); *p* = 0.0048
Basic nursing education	OR = 0.69 (0.43; 1.11); *p* = 0.1253	OR = 0.70 (0.44; 1.11); *p* = 0.1268	OR = 0.95 (0.59; 1.52); *p* = 0.8240	OR = 0.71 (0.43; 1.18); *p* = 0.1893
Bachelor	OR = 1.27 (0.78; 2.06); *p* = 0.3388	OR = 1.01 (0.62; 1.65); *p* = 0.9642	OR = 0.93 (0.55; 1.56); *p* = 0.7851	OR = 1.42 (0.86; 2.34); *p* = 0.1669
WHR	OR = 1.21 (0.81; 1.78); *p* = 0.3515	OR = 1.39 (0.94; 2.05); *p* = 0.1000	OR = 1.33 (0.89; 2.00); *p* = 0.1652	OR = 1.22 (0.81; 1.84); *p* = 0.3362
FAT excessive body fat	OR = 1.74 (1.07; 2.81); *p* = 0.0244	OR = 1.20 (0.74; 1.96); *p* = 0.4602	OR = 1.71 (1.05; 2.79); *p* = 0.0314	OR = 1.71 (1.04; 2.81); *p* = 0.0332
FAT obesity	OR = 1.13 (0.57; 2.23); *p* = 0.7242	OR = 1.69 (0.89; 3.20); *p* = 0.1105	OR = 0.80 (0.38; 1.70); *p* = 0.5682	OR = 1.41 (0.71; 2.78); *p* = 0.3279
BMI	OR = 1.98 (1.31; 2.99); *p* = 0.0011	OR = 1.81 (1.21; 2.71); *p* = 0.0041	OR = 1.85 (1.21; 2.83); *p* = 0.0043	OR = 2.00 (1.3; 3.080); *p* = 0.0017
Diseases	OR = 1.67 (1.12; 2.48); *p* = 0.0111	OR = 1.44 (0.98; 2.13); *p* = 0.0652	OR = 0.88 (0.59; 1.32); *p* = 0.5374	OR = 1.68 (1.11; 2.54); *p* = 0.0133
Age	OR = 0.99 (0.67; 1.47); *p* = 0.9728	OR = 1.09 (0.73; 1.61); *p* = 0.6800	OR = 1.56 (1.03; 2.35); *p* = 0.0367	OR = 1.06 (0.70; 1.60); *p* = 0.7821
FFM	OR = 1.70 (1.13; 2.55); *p* = 0.0106	OR = 1.86 (1.24; 2.78); *p* = 0.0026	OR = 1.16 (0.76; 1.78); *p* = 0.4814	OR = 1.43 (0.94; 2.19); *p* = 0.0960
SBP	OR = 1.04 (0.70; 1.54); *p* = 0.8539	OR = 1.04 (0.71; 1.55); *p* = 0.8279	OR = 0.72 (0.48; 1.08); *p* = 0.1143	OR = 0.90 (0.60; 1.36); *p* = 0.6223
DBP	OR = 0.78 (0.53; 1.17); *p* = 0.2329	OR = 0.73 (0.49; 1.08); *p* = 0.1116	OR = 0.68 (0.45; 1.02); *p* = 0.0650	OR = 0.79 (0.52; 1.19); *p* = 0.2549

* The table presents adjusted model results for the “high” category of the My Eating Habits (MEH) scale and its subscales (Factor I—Habitual Overeating, Factor II—Emotional Overeating, Factor III—Dietary Restriction). Results for the “medium” and “low” categories are provided in Supplementary File, Part II. All models were adjusted using the same set of covariates. Odds ratios (OR) were calculated assuming the following reference groups: 0—female, work in a hospital ward, single-shift work system, full-time job, education—master’s degree, WHR—normal, FAT—normal, BMI—normal, no diseases, age below 45.5 years, FFM below 50.8, SBP below 119.2, DBP below 73.8. For selected variables: 1—secondary medical education, FAT—increased, 2—bachelor’s degree, FAT—excessive.

## Data Availability

All data generated or analyzed during this study have been included in this published article (and its Supplementary Files). Additional information is available from the corresponding author upon reasonable request due to privacy and ethical restrictions, as the dataset contains sensitive personal information of participants.

## Supplementary Materials

The following supporting information can be downloaded at https://www.mdpi.com/article/10.3390/nu17162686/s1, Part I—Tables S1–S3 present all factors influencing the eating habits of nurses in factor 1, factor 2 and factor 3. Part II—Tables S1–S4 present the odds ratios for high, average, and low overall eating habit scores, as well as for the three subscales corresponding to factor 1, factor 2, and factor 3 in the studied group.

## Abbreviations

The following abbreviations are used in this manuscript:

BMI	Body Mass Index
FFM	Fat-Free Mass
FATM	Fat Mass
FATP	Body Fat Percentage
SBP	Systolic Blood Pressure
DBP	Diastolic Blood Pressure
MEH	My Eating Habits
WHO	World Health Organization
OECD	Organisation for Economic Co-operation and Development
WHR	Waist-to-Hip Ratio
ROC	Receiver Operating Characteristic
M	Mean
Me	Median
SD	Standard Deviation
IQR; Q1–Q3	Interquartile Range
CI	Confidence Interval
CART	Classification and Regression Tree
OR	Odds ratios
