# Supplementary material for: Exploring Body Composition and Eating Habits Among Nurses in Poland
Source: Nutrients. 2025 Aug 20;17(16):2686. doi: 10.3390/nu17162686 (PMC12389092; doi:10.3390/nu17162686)
Supplement: Supplementary file 1 [file nutrients-17-02686-s001.zip › nutrients-3810410-supplementary.pdf]

SUPPLEMENTARY MATERIAL

Manuscript title: *Exploring Body Composition and Eating Habits among Nurses in Poland*

Manuscript ID: nutrients-3810410

TABLE OF CONTENTS

1. **STROBE Statement - Checklist** *(pages 1-4)*
2. **SUPPLEMENTARY PART I** *(pages 5-7)* – Tables present all factors influencing the eating habits of nurses in factor 1, factor 2 and factor 3.

- Table S-1. Variables influencing factor 1 of eating habits - Habitual overeating in the studied group of nurses
- Table S-2. Variables influencing factor 2 of eating habits - Emotional overeating in the studied group of nurses
- Table S-3. Variables influencing factor 3 of eating habits - Dietary restrictions in the studied group of nurses

3. **SUPPLEMENTARY PART II** *(pages 7-10)* – Tables present the odds ratios for high, average, and low overall eating habit scores, as well as for the three subscales corresponding to factor 1, factor 2, and factor 3 in the studied group

- Table S-1. Odds ratios for factor 1 - habitual overeating in the studied group
- Table S-2. Odds ratios for factor 2 - emotional overeating in the studied group
- Table S-3. Odds ratios for factor 3 – dietary restrictions in the studied group
- Table S-4. Odds ratios for overall dietary habits in the studied group

Ad. 1. STROBE Statement - Checklist of items that should be included in reports of *cross-sectional studies*

	Item No	Recommendation	Page/Section in manuscript
**Title and abstract**	1	(*a*) Indicate the study's design with a commonly used term in the title or the abstract	Page 1, line 22 (abstract)
		(*b*) Provide in the abstract an informative and balanced summary of what was done and what was found	Page 1, line 28-38
**Introduction**			

Background/rationale	2	Explain the scientific background and rationale for the investigation being reported	Page 2, line 45-91 (introduction)
Objectives	3	State specific objectives, including any prespecified hypotheses	Page 2, line 92-100
**Methods**			
Study design	4	Present key elements of study design early in the paper	Page 3, subsection Participants and study design
Setting	5	Describe the setting, locations, and relevant dates, including periods of recruitment, exposure, follow-up, and data collection	Page 3, subsection Participants and study design
Participants	6	(*a*) Give the eligibility criteria, and the sources and methods of selection of participants	Page 3, subsection Participants and study design
Variables	7	Clearly define all outcomes, exposures, predictors, potential confounders, and effect modifiers. Give diagnostic criteria, if applicable	Page 4 I 5, subsection Measurements and Questionnaire
Data sources/ measurement	8*	For each variable of interest, give sources of data and details of methods of assessment (measurement). Describe comparability of assessment methods if there is more than one group	**Page 4, lines 130-206, methodology section: Measurements and questionnaire.** **No comparison groups were used.** *Not applicable – no exposed/unexposed groups were defined.
Bias	9	Describe any efforts to address potential sources of bias	**Page 4, lines 130-206, Methodology section (Measurements and questionnaire).** Self-reported data acknowledged as potential source of bias; this is addressed in limitations section.
Study size	10	Explain how the study size was arrived at	Page 3, line 121-127.
Quantitative variables	11	Explain how quantitative variables were handled in the analyses. If	Page 5, subsection statistical analysis

		applicable, describe which groupings were chosen and why	
Statistical methods	12	(*a*) Describe all statistical methods, including those used to control for confounding	Page 5, subsection statistical analysis
		(*b*) Describe any methods used to examine subgroups and interactions	Page 5, subsection statistical analysis
		(*c*) Explain how missing data were addressed	Page 5, subsection statistical analysis
		(*d*) If applicable, describe analytical methods taking account of sampling strategy	Page 5, subsection statistical analysis
		(*e*) Describe any sensitivity analyses	Page 5, subsection statistical analysis
**Results**			
Participants	13*	(a) Report numbers of individuals at each stage of study—eg numbers potentially eligible, examined for eligibility, confirmed eligible, included in the study, completing follow-up, and analysed	Table 1, Characteristics of study group
		(b) Give reasons for non-participation at each stage	Not applicable
		(c) Consider use of a flow diagram	Not applicable
Descriptive data	14*	(a) Give characteristics of study participants (eg demographic, clinical, social) and information on exposures and potential confounders	Table 1, Characteristics of study group
		(b) Indicate number of participants with missing data for each variable of interest	Not applicable
Outcome data	15*	Report numbers of outcome events or summary measures	Results section, page 5-16
Main results	16	(*a*) Give unadjusted estimates and, if applicable, confounder-adjusted estimates and their precision (eg, 95% confidence interval). Make clear which confounders were	Results section, page 5-16

		adjusted for and why they were included	
		(*b*) Report category boundaries when continuous variables were categorized	Results section, page 5-16
		(*c*) If relevant, consider translating estimates of relative risk into absolute risk for a meaningful time period	Not applicable
Other analyses	17	Report other analyses done—eg analyses of subgroups and interactions, and sensitivity analyses	Results section, page 5-16
**Discussion**			
Key results	18	Summarise key results with reference to study objectives	Page 16-19, discussion section
Limitations	19	Discuss limitations of the study, taking into account sources of potential bias or imprecision. Discuss both direction and magnitude of any potential bias	Page 19, line 659-668
Interpretation	20	Give a cautious overall interpretation of results considering objectives, limitations, multiplicity of analyses, results from similar studies, and other relevant evidence	Page 16-19, discussion section
Generalisability	21	Discuss the generalisability (external validity) of the study results	Page 19, line 718-722
**Other information**			
Funding	22	Give the source of funding and the role of the funders for the present study and, if applicable, for the original study on which the present article is based	Not applicable

*Give information separately for exposed and unexposed groups.

AD. 1. SUPPLEMENTARY PART I – Tables S-1, S-2 and S-3 present all factors influencing the eating habits of nurses in factor 1, factor 2 and factor 3.

Table S-1. Variables influencing factor 1 of eating habits - Habitual overeating in the studied group of nurses

**Varibles**		**Factor 1 - Habitual overeating**						**p**
		high		low		average		
		N	%	N	%	N	%	
Sex	female	136	32,4%	156	37,1%	128	30,5%	$\chi2$=0,554; p($\chi2$)=0,7582
	male	11	27,5%	17	42,5%	12	30,0%	
Type of work	hospital ward	124	32,1%	150	38,9%	112	29,0%	$\chi2$=2,608; p($\chi2$)=0,2714
	administrative position	23	31,1%	23	31,1%	28	37,8%	
Work system	one shiftwork	60	26,9%	103	46,2%	60	26,9%	$\chi2$=13,698; p($\chi2$)=0,0011
	shift work and night duty	87	36,7%	70	29,5%	80	33,8%	
More than one job	no	90	27,7%	132	40,6%	103	31,7%	$\chi2$=9,539; p($\chi2$)=0,0085
	yes	57	42,2%	41	30,4%	37	27,4%	
Education	basic nursing education	35	25,4%	50	36,2%	53	38,4%	$\chi2$=7,885; p($\chi2$)=0,0959
	Bachelor	40	38,5%	37	35,6%	27	26,0%	
	Master degree	72	33,0%	86	39,4%	60	27,5%	
WHR	normal	73	30,0%	93	38,3%	77	31,7%	$\chi2$=0,917; p($\chi2$)=0,6322
	Abdominal obesity	74	34,1%	80	36,9%	63	29,0%	
BMI	normal	95	29,2%	133	40,9%	97	29,8%	$\chi2$=9,510; p($\chi2$)=0,0495
	increased	38	41,8%	22	24,2%	31	34,1%	
	excessive	14	31,8%	18	40,9%	12	27,3%	
BMI	no overweight/obesity	47	23,7%	92	46,5%	59	29,8%	$\chi2$=14,645; p($\chi2$)=0,0007
	prevalence overweight/obesity	100	38,2%	81	30,9%	81	30,9%	
Prevalence of desease	no	63	26,6%	99	41,8%	75	31,6%	$\chi2$=6,907; p($\chi2$)=0,0316
	yes	84	37,7%	74	33,2%	65	29,1%	

Table S-2. Variables influencing factor 2 of eating habits - Emotional overeating in the studied group of nurses

**Varibles**		**Factor 2 - Emotional overeating**						**p**
		high		average		low		
		N	%	N	%	N	%	
Sex	female	143	34,0%	62	14,8%	215	51,2%	

	male	9	22,5%	9	22,5%	22	55,0%	χ2=2,994; p(χ2)=0,2239
Type of work	hospital ward	129	33,4%	57	14,8%	200	51,8%	χ2=0,836; p(χ2)=0,6584
	administrative position	23	31,1%	14	18,9%	37	50,0%	
Work system	one shiftwork	65	29,1%	40	17,9%	118	52,9%	χ2=3,907; p(χ2)=0,1418
	shift work and night duty	87	36,7%	31	13,1%	119	50,2%	
More than one job	no	106	32,6%	43	13,2%	176	54,2%	χ2=5,036; p(χ2)=0,0806
	yes	46	34,1%	28	20,7%	61	45,2%	
Education	basic nursing education	38	27,5%	20	14,5%	80	58,0%	χ2=4,306; p(χ2)=0,3662
	Bachelor	37	35,6%	19	18,3%	48	46,2%	
	Master degree	77	35,3%	32	14,7%	109	50,0%	
WHR	normal	72	29,6%	41	16,9%	130	53,5%	χ2=2,897; p(χ2)=0,2349
	Abdominal obesity	80	36,9%	30	13,8%	107	49,3%	
BMI	normal	101	31,1%	49	15,1%	175	53,8%	F=5,256; p(F)=0,2605
	increased	32	35,2%	18	19,8%	41	45,1%	
	excessive	19	43,2%	4	9,1%	21	47,7%	
BMI	no overweight/obesity	51	25,8%	31	15,7%	116	58,6%	χ2=8,963; p(χ2)=0,0113
	prevalence overweight/obesity	101	38,5%	40	15,3%	121	46,2%	
Prevalence of desease	no	69	29,1%	37	15,6%	131	55,3%	χ2=3,631; p(χ2)=0,1628
	yes	83	37,2%	34	15,2%	106	47,5%	

Table S-3. Variables influencing factor 3 of eating habits - Dietary restrictions in the studied group of nurses

Varibles		Factor 3 - Dietary restrictions						p
		low		high		average		
		N	%	N	%	N	%	
Sex	female	167	39,8%	124	29,5%	129	30,7%	χ2=1,760; p(χ2)=0,4148
	male	17	42,5%	8	20,0%	15	37,5%	
Type of work	hospital ward	155	40,2%	111	28,8%	120	31,1%	χ2=0,053; p(χ2)=0,9736
	administrative position	29	39,2%	21	28,4%	24	32,4%	
Work system	one shiftwork	90	40,4%	60	26,9%	73	32,7%	χ2=0,78; p(χ2)=0,6770
	shift work and night duty	94	39,7%	72	30,4%	71	30,0%	
More than one job	no	140	43,1%	85	26,2%	100	30,8%	χ2=5,216; p(χ2)=0,0737
	yes	44	32,6%	47	34,8%	44	32,6%	
Education	basic nursing education	63	45,7%	39	28,3%	36	26,1%	

	Bachelor	33	31,7%	29	27,9%	42	40,4%	χ2=6,967; p(χ2)=0,1377
	Master degree	88	40,4%	64	29,4%	66	30,3%	
WHR	normal	103	42,4%	63	25,9%	77	31,7%	χ2=2,135; p(χ2)=0,3439
	Abdominal obesity	81	37,3%	69	31,8%	67	30,9%	
BMI	normal	143	44,0%	87	26,8%	95	29,2%	χ2=11,156; p(χ2)=0,0249
	increased	26	28,6%	35	38,5%	30	33,0%	
	excessive	15	34,1%	10	22,7%	19	43,2%	
BMI	no overweight/obesity	100	50,5%	43	21,7%	55	27,8%	χ2=16,872; p(χ2)=0,0002
	prevalence overweight/obesity	84	32,1%	89	34,0%	89	34,0%	
Prevalence of desease	no	102	43,0%	71	30,0%	64	27,0%	χ2=4,287; p(χ2)=0,1172
	yes	82	36,8%	61	27,4%	80	35,9%	

AD. 2. SUPPLEMENTARY PART II – Tables S-1, S-2, S-3, and S-4 present the odds ratios for high, average, and low overall eating habit scores, as well as for the three subscales corresponding to factor 1, factor 2, and factor 3 in the studied group.

Table S-1. Odds ratios for factor 1 - habitual overeating in the studied group

Variables	High	Average	Low
Sex	OR=0,79 (0,38;1,63); p=0,5278	OR=0,98 (0,48;1,98); p=0,9501	OR=1,25 (0,65;2,41); p=0,5046
Type of work	OR=0,95 (0,56;1,63); p=0,8601	OR=1,49 (0,89;2,50); p=0,1324	OR=0,71 (0,42;1,21); p=0,2071
Work system	OR=1,58 (1,06;2,34); p=0,0247	OR=1,38 (0,93;2,07); p=0,1112	OR=0,49 (0,33;0,72); p=0,0003
More than one job	OR=1,91 (1,25;2,90); p=0,0025	OR=0,81 (0,52;1,27); p=0,3635	OR=0,64 (0,42;0,98); p=0,0396
Basic nursing education	OR=0,69 (0,43;1,11); p=0,1253	OR=1,64 (1,04;2,59); p=0,0322	OR=0,87 (0,56;1,36); p=0,5428
Bachelor	OR=1,27 (0,78;2,06); p=0,3388	OR=0,92 (0,54;1,57); p=0,7680	OR=0,85 (0,52;1,38); p=0,5038
WHR	OR=1,21 (0,81;1,78); p=0,3515	OR=0,88 (0,59;1,31); p=0,5368	OR=0,94 (0,65;1,37); p=0,7561
FAT excessive body fat	OR=1,74 (1,07;2,81); p=0,0244	OR=1,21 (0,74;1,99); p=0,4412	OR=0,46 (0,27;0,78); p=0,0040
FAT obesity	OR=1,13 (0,57;2,23); p=0,7242	OR=0,88 (0,44;1,78); p=0,7256	OR=1,00 (0,53;1,90); p=0,9986
BMI	OR=1,98 (1,31;2,99); p=0,0011	OR=1,05 (0,71;1,58); p=0,7964	OR=0,52 (0,35;0,76); p=0,0007
Deseases	OR=1,67 (1,12;2,48); p=0,0111	OR=0,89 (0,60;1,32); p=0,5608	OR=0,69 (0,47;1,01); p=0,0578
Age	OR=0,99 (0,67;1,47); p=0,9728	OR=1,07 (0,72;1,60); p=0,7297	OR=0,94 (0,65;1,38); p=0,7677
FFM	OR=1,70 (1,13;2,55); p=0,0106	OR=0,77 (0,50;1,19); p=0,2415	OR=0,76 (0,50;1,13); p=0,1756

SBP	OR=1,04 (0,70;1,54); p=0,8539	OR=0,81 (0,54;1,20); p=0,2947	OR=1,17 (0,80;1,72); p=0,4130
DBP	OR=0,78 (0,53;1,17); p=0,2329	OR=1,06 (0,71;1,60); p=0,7731	OR=1,19 (0,81;1,75); p=0,3815

Odds rations (OR) were calculated assuming the following reference groups: 0 – female, work in a hospital ward, single-shift work system, full-time job, education: master's degree, WHR – normal, FAT – normal, BMI – normal, no diseases, age below 45.5 years, FFM below 50.8, SBP below 119.2, DBP below 73.8. For selected variables: 1 – secondary medical education, FAT – increased; 2 – bachelor's degree, FAT – excessive.

Table S-2. Odds ratios for factor 2 - emotional overeating in the studied group

Variables	High	Average	Low
Sex	OR=0,56 (0,26;1,21); p=0,1424	OR=1,68 (0,76;3,69); p=0,1997	OR=1,17 (0,61;2,24); p=0,6453
Type of work	OR=0,90 (0,53;1,54); p=0,6953	OR=1,35 (0,71;2,57); p=0,3665	OR=0,93 (0,57;1,53); p=0,7750
Work system	OR=1,41 (0,95;2,09); p=0,0854	OR=0,69 (0,41;1,15); p=0,1510	OR=0,90 (0,62;1,29); p=0,5620
More than one job	OR=1,07 (0,70;1,63); p=0,7620	OR=1,72 (1,01;2,90); p=0,0439	OR=0,70 (0,47;1,04); p=0,0803
Basic nursing education	OR=0,70 (0,44;1,11); p=0,1268	OR=0,99 (0,54;1,80); p=0,9614	OR=1,38 (0,90;2,12); p=0,1425
Bachelor	OR=1,01 (0,62;1,65); p=0,9642	OR=1,30 (0,70;2,42); p=0,4101	OR=0,86 (0,54;1,37); p=0,5186
WHR	OR=1,39 (0,94;2,05); p=0,1000	OR=0,79 (0,47;1,32); p=0,3671	OR=0,85 (0,59;1,22); p=0,3696
FAT excessive body fat	OR=1,20 (0,74;1,96); p=0,4602	OR=1,39 (0,76;2,53); p=0,2821	OR=0,70 (0,44;1,12); p=0,1389
FAT obesity	OR=1,69 (0,89;3,20); p=0,1105	OR=0,56 (0,19;1,65); p=0,2939	OR=0,78 (0,42;1,47); p=0,4461
BMI	OR=1,81 (1,21;2,71); p=0,0041	OR=0,97 (0,58;1,62); p=0,9089	OR=0,61 (0,42;0,88); p=0,0086
Deseases	OR=1,44 (0,98;2,13); p=0,0652	OR=0,97 (0,59;1,61); p=0,9137	OR=0,73 (0,51;1,06); p=0,0972
Age	OR=1,09 (0,73;1,61); p=0,6800	OR=0,86 (0,52;1,43); p=0,5675	OR=1,00 (0,70;1,45); p=0,9798
FFM	OR=1,86 (1,24;2,78); p=0,0026	OR=1,07 (0,63;1,82); p=0,8016	OR=0,55 (0,37;0,81); p=0,0026
SBP	OR=1,04 (0,71;1,55); p=0,8279	OR=0,99 (0,60;1,65); p=0,9729	OR=0,97 (0,67;1,40); p=0,8571
DBP	OR=0,73 (0,49;1,08); p=0,1116	OR=1,44 (0,84;2,47); p=0,1806	OR=1,11 (0,76;1,61); p=0,5973

Odds rations (OR) were calculated assuming the following reference groups: 0 – female, work in a hospital ward, single-shift work system, full-time job, education: master's degree, WHR – normal, FAT – normal, BMI – normal, no diseases, age below 45.5 years, FFM below 50.8, SBP below 119.2, DBP below 73.8. For selected variables: 1 – secondary medical education, FAT – increased; 2 – bachelor's degree, FAT – excessive.

Table S-3. Odds ratios for factor 3 – dietary restrictions in the studied group

Variables	High	Average	Low
Sex	OR=0,60 (0,27;1,33); p=0,2075	OR=1,35 (0,69;2,65); p=0,3779	OR=1,12 (0,58;2,16); p=0,7356
Type of work	OR=0,98 (0,57;1,70); p=0,9475	OR=1,06 (0,62;1,81); p=0,8193	OR=0,96 (0,58;1,60); p=0,8765

Work system	OR=1,19 (0,79;1,78); p=0,4106	OR=0,88 (0,59;1,30); p=0,5210	OR=0,97 (0,67;1,41); p=0,8789
More than one job	OR=1,51 (0,98;2,32); p=0,0623	OR=1,09 (0,71;1,67); p=0,7010	OR=0,64 (0,42;0,97); p=0,0373
Basic nursing education	OR=0,95 (0,59;1,52); p=0,8240	OR=0,81 (0,50;1,31); p=0,3948	OR=1,24 (0,81;1,91); p=0,3258
Bachelor	OR=0,93 (0,55;1,56); p=0,7851	OR=1,56 (0,96;2,54); p=0,0733	OR=0,69 (0,42;1,12); p=0,1355
WHR	OR=1,33 (0,89;2,00); p=0,1652	OR=0,96 (0,65;1,43); p=0,8514	OR=0,81 (0,56;1,18); p=0,2691
FAT excessive body fat	OR=1,71 (1,05;2,79); p=0,0314	OR=1,19 (0,72;1,96); p=0,4923	OR=0,51 (0,31;0,84); p=0,0088
FAT obesity	OR=0,80 (0,38;1,70); p=0,5682	OR=1,84 (0,97;3,50); p=0,0629	OR=0,66 (0,34;1,27); p=0,2149
BMI	OR=1,85 (1,21;2,83); p=0,0043	OR=1,34 (0,89;2,00); p=0,1568	OR=0,46 (0,32;0,68); p=0,0001
Deseases	OR=0,88 (0,59;1,32); p=0,5374	OR=1,51 (1,02;2,25); p=0,0408	OR=0,77 (0,53;1,12); p=0,1707
Age	OR=1,56 (1,03;2,35); p=0,0367	OR=0,68 (0,46;1,01); p=0,0550	OR=0,98 (0,67;1,42); p=0,9086
FFM	OR=1,16 (0,76;1,78); p=0,4814	OR=1,37 (0,91;2,07); p=0,1287	OR=0,65 (0,44;0,97); p=0,0371
SBP	OR=0,72 (0,48;1,08); p=0,1143	OR=1,85 (1,23;2,80); p=0,0032	OR=0,77 (0,53;1,13); p=0,1793
DBP	OR=0,68 (0,45;1,02); p=0,0650	OR=1,18 (0,79;1,78); p=0,4141	OR=1,20 (0,82;1,76); p=0,3504

Odds rations (OR) were calculated assuming the following reference groups: 0 – female, work in a hospital ward, single-shift work system, full-time job, education: master's degree, WHR – normal, FAT – normal, BMI – normal, no diseases, age below 45.5 years, FFM below 50.8, SBP below 119.2, DBP below 73.8. For selected variables: 1 – secondary medical education, FAT – increased; 2 – bachelor's degree, FAT – excessive.

Table S-4. Odds ratios for overall dietary habits in the studied group

Variables	High	Average	Low
Sex	OR=0,85 (0,40;1,80); p=0,6766	OR=0,86 (0,41;1,77); p=0,6732	OR=1,29 (0,67;2,48); p=0,4403
Type of work	OR=1,03 (0,59;1,79); p=0,9079	OR=1,39 (0,82;2,34); p=0,2181	OR=0,72 (0,43;1,21); p=0,2098
Work system	OR=1,55 (1,03;2,35); p=0,0370	OR=0,81 (0,54;1,21); p=0,2986	OR=0,84 (0,58;1,22); p=0,3520
More than one job	OR=1,86 (1,21;2,87); p=0,0048	OR=0,81 (0,52;1,27); p=0,3635	OR=0,69 (0,46;1,05); p=0,0837
Basic nursing education	OR=0,71 (0,43;1,18); p=0,1893	OR=0,88 (0,55;1,40); p=0,5950	OR=1,44 (0,93;2,21); p=0,0994
Bachelor	OR=1,42 (0,86;2,34); p=0,1669	OR=0,92 (0,55;1,52); p=0,7382	OR=0,78 (0,48;1,27); p=0,3218
WHR	OR=1,22 (0,81;1,84); p=0,3362	OR=0,96 (0,64;1,43); p=0,8323	OR=0,88 (0,61;1,28); p=0,4985
FAT excessive body fat	OR=1,71 (1,04;2,81); p=0,0332	OR=1,01 (0,61;1,68); p=0,9551	OR=0,61 (0,37;1,00); p=0,0478
FAT obesity	OR=1,41 (0,71;2,78); p=0,3279	OR=0,96 (0,48;1,91); p=0,9013	OR=0,78 (0,41;1,49); p=0,4540
BMI	OR=2,00 (1,3;3,080); p=0,0017	OR=1,10 (0,74;1,64); p=0,6436	OR=0,53 (0,36;0,77); p=0,0010
Deseases	OR=1,68 (1,11;2,54); p=0,0133	OR=0,96 (0,65;1,44); p=0,8601	OR=0,67 (0,46;0,98); p=0,0364

Age	OR=1,06 (0,70;1,60); p=0,7821	OR=0,87 (0,59;1,30); p=0,5009	OR=1,07 (0,74;1,56); p=0,7062
FFM	OR=1,43 (0,94;2,19); p=0,0960	OR=1,47 (0,97;2,22); p=0,0688	OR=0,52 (0,34;0,77); p=0,0014
SBP	OR=0,90 (0,60;1,36); p=0,6223	OR=0,91 (0,61;1,36); p=0,6633	OR=1,18 (0,81;1,71); p=0,3932
DBP	OR=0,79 (0,52;1,19); p=0,2549	OR=0,86 (0,57;1,28); p=0,4547	OR=1,40 (0,96;2,06); p=0,0835

Odds rations (OR) were calculated assuming the following reference groups: 0 – female, work in a hospital ward, single-shift work system, full-time job, education: master's degree, WHR – normal, FAT – normal, BMI – normal, no diseases, age below 45.5 years, FFM below 50.8, SBP below 119.2, DBP below 73.8. For selected variables: 1 – secondary medical education, FAT – increased; 2 – bachelor's degree, FAT – excessive.
